# Vaccine-induced Aβ-specific CD8^+^ T cells do not trigger autoimmune neuroinflammation in a murine model of Alzheimer’s disease

**DOI:** 10.1186/s12974-015-0317-5

**Published:** 2015-05-16

**Authors:** Martine Bruley Rosset, Gabrielle Lui, Cira Dansokho, Thomas Chaigneau, Guillaume Dorothée

**Affiliations:** INSERM, UMR_S 938, CdR Saint-Antoine, Laboratory Immune System, Neuroinflammation and Neurodegenerative Diseases, Inflammation-Immunopathology-Biotherapy Department (DHU i2B), Hôpital Saint-Antoine, 184 rue du Faubourg Saint-Antoine, F-75012 Paris, France; Sorbonne Universités, UPMC Univ Paris 06, UMR_S 938, CdR Saint-Antoine, Hôpital Saint-Antoine, 184 rue du Faubourg Saint-Antoine, F-75012 Paris, France

**Keywords:** Alzheimer’s disease, Vaccination, Aβ peptide, CD8^+^ T cells, Encephalitis

## Abstract

**Background:**

Active immunization against Aβ was reported to have a therapeutic effect in murine models of Alzheimer’s disease. Clinical Aβ vaccination trial AN1792 was interrupted due to the development in 6 % of the patients of meningoencephalitis likely involving pro-inflammatory CD4^+^ T cells. However, the potential implication of auto-aggressive anti-Aβ CD8^+^ T cells has been poorly investigated.

**Methods:**

Potential MHC-I-restricted Aβ-derived epitopes were first analyzed for their capacity to recruit functional CD8^+^ T cell responses in mouse models. Their impact on migration of CD8^+^ T cells into the brain parenchyma and potential induction of meningoencephalitis and/or neuronal damage was investigated upon vaccination in the APPPS1 mouse model of AD.

**Results:**

We identified one nonamer peptide, Aβ33-41, which was naturally processed and presented in association with H-2-D^b^ molecule on neurons and CD11b^+^ microglia. Upon optimization of anchor residues for enhanced binding to H-2-D^b^, immunization with the modified Aβ33-41NP peptide elicited Aβ-specific IFNγ-secreting CD8^+^ T cells, which are cytotoxic towards Aβ-expressing targets. Whereas T cell infiltration in the brain of APPPS1 mice is dominated by CD3^+^CD8^−^ T cells and increases with disease evolution between 4 and 7 months of age, a predominance of CD3^+^CD8^+^ over CD3^+^CD8^−^ cells was observed in 6- to 7-month-old APPPS1 but not in WT animals, only after vaccination with Aβ33-41NP. The number of CD11b^+^ mononuclear phagocytes, which significantly increases with age in the brain of APPPS1 mice, was reduced following immunization with Aβ33-41NP. Despite peripheral activation of Aβ-specific CD8^+^ cytotoxic effectors and enhanced infiltration of CD8^+^ T cells in the brain of Aβ33-41NP-immunized APPPS1 mice, no clinical signs of severe autoimmune neuroinflammation were observed.

**Conclusions:**

Altogether, these results suggest that Aβ-specific CD8^+^ T cells are not major contributors to meningoencephalitis in response to Aβ vaccination.

## Background

Alzheimer’s disease (AD) is a severe neurodegenerative disorder characterized by progressive loss of memory and cognitive functions. Accumulation of amyloid-β peptide (Aβ), both as soluble oligomers and in the form of amyloid plaques, plays a key role in initiating the pathogenic cascade in AD. Induction of specific adaptive immune responses following vaccination against Aβ1-42 was reported to be of benefit in animal models of AD [1–3]. Antibodies to Aβ1-42 were proved efficient in clearing amyloid plaques and in attenuating pathology in mouse models of the disease [4, 5]. In line with these encouraging results, clinical vaccination trial (AN1792) was initiated and, although modest beneficial effects were observed in a cohort of Aβ1-42-immunized AD patients, 6 % of them developed meningoencephalitis [6]. Postmortem neuropathological examination carried out in few Aβ-vaccinated patients who developed such severe side effects revealed the presence of CD4^+^ and/or CD8^+^ T cells, together with extensive macrophage infiltration and a reduction in amyloid burden [7, 8]. Second-generation immunotherapy trials that rely exclusively on Aβ-specific antibodies were then initiated, based either on the intravenous infusion of humanized anti-Aβ monoclonal antibodies (mAbs) or vaccination strategies using the N-terminal portion of Aβ to elicit antibodies without inducing Aβ-specific T cell responses. Among these strategies, trials with bapineuzumab [9, 10] and solanezumab [11] were the most advanced, but results of phase 3 studies released in summer 2012 indicated that both antibodies were inefficient at improving cognitive performance in AD patients. Failure of these trials underlined the need for readdressing the therapeutic capability of antibodies alone, and the role of T cells in the therapeutic effect and/or meningoencephalitis observed in initial immunization studies based on vaccination with full-length Aβ.

Increased T cell reactivity to Aβ has been observed in the blood of elderly subjects and patients with AD, and various Aβ-derived HLA class II-restricted CD4^+^ T cell epitopes have been described [12]. Different HLA-DR alleles were shown to impact Aβ immunogenicity in humans and were associated with Aβ-specific T cell responses to distinct T cell epitopes within residues 15–42 of Aβ [13]. Aβ-derived epitopes eliciting CD4^+^ T cell responses have also been identified in different mouse haplotypes and HLA class II transgenic mice [14–17]. Immunization of F1 SJLxB6 amyloid precursor protein (APP) transgenic (APP-Tg) AD mice with dominant T cell epitope Aβ10-24 stimulated peripheral CD4^+^ T cell responses but did not result in T cell infiltration nor the occurrence of meningoencephalitis. However, enhanced expression of IFNγ in the brain of APP-Tg mice promotes T cell infiltration targeted primarily to the sites of Aβ deposition and was associated with both clearance of Aβ and transient encephalitis upon immunization with Aβ10-24, in the absence of antibodies to Aβ [18]. Altogether, these reports suggest that the magnitude of Aβ-specific CD4^+^ T cell responses critically depends on the nature of Aβ-derived T cell epitopes, which significantly vary with MHC genotype. Thus, inappropriate boosting of endogenous naturally occurring Aβ-specific CD4^+^ T cell responses, e.g. through vaccination in the presence of a Th1 adjuvant, may likely be involved in the development of meningoencephalitis in selected AN1792 patients displaying given MHC haplotypes.

Besides CD4^+^ T cell responses to Aβ, vaccination with full-length Aβ1-42 may also activate Aβ-specific CD8^+^ T cells. The characterization of such potentially autoreactive CD8^+^ T cells has received little attention, presumably because of their expected implication in the direct killing of CNS cells and thus reduced therapeutic interest [19]. It was previously reported that vaccination of BALB/c mice with Aβ-encoding DNA elicited IFNγ secretion by splenocytes upon in vitro stimulation with Aβ12-28 but not Aβ10-20, suggesting that a dominant immunogenic epitope lies within amino acids 21 through 28. Reduction of IFNγ secretion upon depletion of CD8^+^ T cells identified the phenotype of these effectors [15]. Nevertheless, the potential impact of such MHC-I-restricted Aβ-specific T cell responses on disease evolution and the development of meningoencephalitis upon immunization remains unknown.

In order to evaluate the role of Aβ-specific CD8^+^ T cells in a mouse model of AD, we aimed at identifying MHC class I-restricted Aβ-derived epitopes able to recruit functional CD8^+^ T cell responses in C57BL/6 mice. Vaccination with such epitopes in the absence of concomitant Aβ-specific humoral nor CD4^+^ T cell responses was used to analyze the migration of CD8^+^ T cells into the brain parenchyma of APPPS1 mice, and their potential role in the induction of meningoencephalitis and/or neuronal damage. We identified one H-2-D^b^-restricted naturally processed peptide, Aβ33-41, which elicits Aβ-specific IFNγ-secreting CD8^+^ T cells displaying in vitro cytotoxic activity towards Aβ-expressing targets. Aβ33-41 was naturally processed and presented by CD11b^+^ microglia in the brain of APPPS1 but not WT mice. Despite peripheral activation of functional Aβ-specific CD8^+^ effectors and enhanced CD8^+^ T cell infiltration into the brain parenchyma of APPPS1 mice immunized with anchor-optimized Aβ33-41NP, no clinical signs of autoimmune neuroinflammation were observed.

## Methods

### Mice and ethics statement

C57BL/6 mice (H-2^b^ haplotype) were purchased from Elevage Janvier (Le Genest Saint Isle, France). HLA-A02.01/HLA-DR1-transgenic, H-2^b^ class I-/II-knockout mice [20, 21] on the C57BL/B6 background were kindly provided by Pr François Lemonnier (Paris, France) and were bred in our animal facility. APPPS1 double transgenic mice (Thy1-APP^KM670/671NL^; Thy1-PS1^L166P^) which overexpress mutated human APP and presenilin 1 genes on the C57BL/6 background were kindly provided by Pr. Mathias Jucker (Tubingen, Germany) [22]. Animals were bred and maintained under strictly monitored specific and opportunistic pathogen-free conditions. All experimental protocols were conducted in accordance with good practices on the care and use of laboratory animals, as defined by the relevant national and/or local animal welfare bodies. Animal studies were approved by the ethics committee of the Alfort School of Veterinary Medicine.

### Plasmids and peptides

A pcDNA3.1 vector encoding the human APP sequence was used for DNA vaccination as already described [23]. Empty pcDNA3.1 plasmid was used as control. Nonamer peptide sequence bearing H-2-D^b^-binding motifs were selected by applying the epitope prediction algorithm BIMAS (www-bimas.cit.nih.gov/) to human APP amino acid sequence. Amino acids were substituted at anchor positions for H-2-D^b^ binding (positions 1, 2, 3, 5, and 9) [24] by amino acids from a high binding affinity peptide from the influenza nucleoprotein (NP366). Modified peptides were mentioned as NP peptides. Native and modified peptides (purity >90 %) were purchased from GeneCust (Dudelange, Luxembourg). Human Aβ1-42 peptide was dissolved at 40 mg/ml in DMSO and stored at −20 °C. Panel of Aβ-derived 9-mer peptides overlapping by three amino acids were dissolved at 3 mM in DMSO, before final dilution in RPMI medium for in vitro T cell stimulation.

### Immunization protocols

For DNA vaccination, mice received two or three intramuscular injections of 100 μg of APP-pcDNA at weekly interval [24]. These mice were sensitized 5 days before with 50 μg cardiotoxin. For Aβ peptide immunization, 6- to 8-week-old mice were immunized by footpad injections with 100 μg of Aβ1-42 emulsified in an equal volume of CFA. In some experiments, mice were boosted with 60 μg of Aβ1-42 emulsified in an equal volume of IFA. When indicated, mice were injected sc at the base of the tail with 100 μg of peptide, 50 μM of oligo-CpG (N°3678), and 100 μg of the I-A^b^-binding hepatitis B virus (HBV) core peptide emulsified in an equal volume of IFA.

### Measurement of relative affinity of peptides for H-2-D^b^

RMAS lymphoma cells are deficient for TAP molecules in charge of endogenous peptide transport and thus express empty and unstable MHC class I molecules. To evaluate the capacity of peptides to bind and stabilize H-2-D^b^ molecules, RMAS cells were incubated for 4 h at 27 °C with various concentrations of peptides. Cells were then washed and cultured for an additional 4-h period at 37 °C in complete culture medium without peptides. For staining, 2 × 10^5^ peptide-loaded RMAS cells were incubated for 20 min at 4 °C with biotin-conjugated H-2-D^b^-specific mAb (clone 28-14-8, BD Biosciences, Le Pont-de-Claix, France), followed by phycoerythrin-conjugated streptavidin. Samples were analyzed with a FACSCalibur (BD) flow cytometer, and data were processed using the FlowJo software (Tree star, San Carlos, CA, USA). Cell-surface fluorescence was expressed as mean fluorescence intensity (MFI).

### Anti-Aβ antibodies measurement by ELISA

To measure serum anti-Aβ antibody titers in immunized mice, flat-bottom Nunc MaxiSorp ELISA plates were coated overnight at 4 °C with Aβ1-42 peptide (1 μg/ml in 0.1 M NaHCO_3_, pH 8.3). Plates were then washed twice with PBS-T and blocked with PBS-1 % BSA for 2 h at room temperature (RT). After two washes, plates were incubated for 2 h at RT with serum samples at various dilutions in blocking buffer. The Aβ1-17-specific mAb 6E10 (0.01 and 1 μg/ml) (Sigma-Aldrich, St Quentin Fallavier, France) was used as a positive internal reference sample. Plates were washed and incubated 90 min at RT with peroxidase-conjugated goat anti-mouse IgG Ab (Amersham, Little Chalfont, UK) and then revealed with O-phenylenediamine/H_2_O_2_ substrate (Sigma-Aldrich). Results were expressed as relative OD = Experimental OD/Reference OD for 6E10 mAb in the same plate.

### FACS analysis

Single-cell suspensions were prepared from the spleens and incubated for 15 min with anti-Fc receptor blocking Ab (2.4G2, BD Biosciences, Franklin Lakes, NJ, USA) to avoid non-specific staining. FITC-conjugated anti-CD4 (L3T4), APC-conjugated anti-CD8 (clone 53-6-7), and PE-conjugated anti-CD19 (clone 1D3) mAbs (all from BD Biosciences) were used for cell-surface staining. Stained cells were analyzed using a FACSCalibur flow cytometer, and data were processed using FlowJo.

### Isolation of adult microglial cells

Brains were dissected, finely minced, and incubated in 5 ml PBS-1X 0.5 % BSA with 0.2 mg/ml Collagenase IV (Roche, France) and 0.5 mg/ml DNase I (Sigma) for 35 min at 37 °C and 5 % CO_2_. After incubation, EDTA (10 mM) was added to the suspension and tissue was mechanically disrupted gently and cell suspensions were passed through a 70-μm cell strainer. Cells were then washed with PBS-1X 0.5 % BSA, resuspended in 4 ml of 30 % percoll solution and transferred into a 15-ml tube. Four ml of 37 % percoll was then added below the cell suspension, followed by 4 ml of 70 % percoll under the previous 37 % layer. Cells were then centrifuged at RT for 40 min at 800 g, supernatant containing the myelin was removed, and interphase layer of mononuclear cells was harvested. Cells were washed with PBS-1X 0.5 % BSA, and microglial cells were further purified using anti-CD11b MicroBeads (Miltenyi Biotec, Paris, France), according to manufacturer’s instructions. Purity of isolated CD11b^+^ cells was >90 %, as verified by flow cytometry.

### ELISPOT assay

Nitrocellulose-based 96-well plates (Millipore, Billerica, MA, USA) were coated with anti-mouse IFN-γ capture mAb (1 μg/ml; clone R4-6A2, BD Biosciences) and saturated with complete culture medium. Responder splenocytes from individual mice were seeded at 10^6^ or 5 × 10^5^ cells/wells and cultured with medium alone or containing 10 μg/ml of peptide. In some experiments, splenocytes were stimulated with CD11b^+^ microglia (3 × 10^4^/wells) isolated from the brain of WT or APPPS1 mice, or with mitomycin-treated 1C11 cells (2 × 10^4^/wells), a neuronal cell line expressing both APP and H-2^b^ MHC-I but not MHC-II molecules [25, 26]. Plates were incubated at 37 °C in 5 % CO_2_ for 18 h, washed, and incubated with biotinylated anti-mouse IFNγ detection mAb (0.5 μ/ml; clone XMG1.2, BD Biosciences). Alkaline phosphatase conjugated to streptavidin (Roche, France) (1/500 dilution, 100 μL/well) was then added. IFNγ-secreting cells were visualized using TNB/BCIP substrate (Promega, Charbonnières, France), and spots were counted using an automatic ELISPOT plate reader. Samples were assayed in triplicate, and the frequency of peptide-specific T cells was calculated after subtracting the mean number of spots obtained in the absence of peptide or cells.

### In vitro ^51^Cr release cytotoxicity assay

Spleen cells (5 × 10^6^ cells/ml) from immunized mice were cultured at 37 °C with the matching NP peptide in complete medium for 5 days. RMAS or 1C11 target cells were labeled with ^51^Cr for 1 h at 37 °C, followed by extensive washes. 10^5^^51^Cr-labeled RMAS cells/wells were then incubated with matching NP peptides (10 μg/ml) for 2 h in triplicate in 96-well round-bottom plates. Immune effectors were then added at various E:T ratios. Alternatively, 2 × 10^4^^51^Cr-labeled 1C11 cells/wells were incubated with immune effectors at various E:T ratios. Culture supernatants were collected 4 h later, and ^51^Cr release (cpm) was measured in a γ counter (LKB, Orsay, France). The percentage of cytotoxicity was calculated as follows: [1- (experimental release–minimum release/maximum release–minimum release)] × 100, where minimum release was the amount of ^51^Cr released by target cells without effectors, and maximum release was the total amount of ^51^Cr obtained after lysis of 10^5^ RMAS or 2 × 10^4^ 1C11 cells.

### Monitoring the percentage of peptide-specific CD8^+^ T cells in immunized mice

Blood was collected at different time points after vaccination, and RBC were lysed. The frequency of 23-31NP- and 33-41NP-specific CD8^+^ T cells was assessed by intracellular IFNγ staining of CD8^+^ cells after 4 h of incubation with either medium alone or with the native or NP peptides, in the presence of brefeldin A (Cytofix/Cytoperm Plus Golgi Plug Kit, BD Biosciences). The percentage of IFNγ^+^ cells among CD8^+^ lymphocytes was evaluated by FACS.

### Immunohistochemistry analyses

Brains collected at different disease stages were frozen in OCT compound (VWR, Leuven, Belgium). The 5-μm cryosections were prepared using a Leica RM2145 microtome and labeled with rabbit anti-mouse CD3 mAb revealed with Alexa Fluor 488-conjugated goat anti-rabbit (Life Technologies), in combination with rat anti-mouse CD8 mAb (Relia Tech Gmb, Braunschweig, Germany) revealed with Alexa Fluor 594-conjugated goat anti-rat mAb. Mononuclear phagocytes were detected using a rat anti-mouse CD11b mAb (eBiosciences, Paris, France) revealed with Alexa Fluor 488-conjugated goat anti-rat mAb. At least three nonadjacent sections from the midbrain area were analyzed, and Dapi-positive stained cells were counted. Numbers were normalized according to the tissue section surface area calculated by the ImageJ software, and three mice per treatment were examined. Amount of hAβ deposition was evaluated using mouse anti-human Aβ mAb (BAM10, Sigma-Aldrich) revealed with Alexa Fluor 594-conjugated goat anti-mouse mAb.

### Statistical analyses

Statistical analyses were carried out with Graphpad Prism software (San Diego, CA, USA), using the Mann–Whitney U test.

## Results

### Humanized HLA-A2.1/HLA-DR1 mice do not constitute an appropriate model for assessing Aβ-specific CD8^+^ T cell responses

**Fig. 1:**
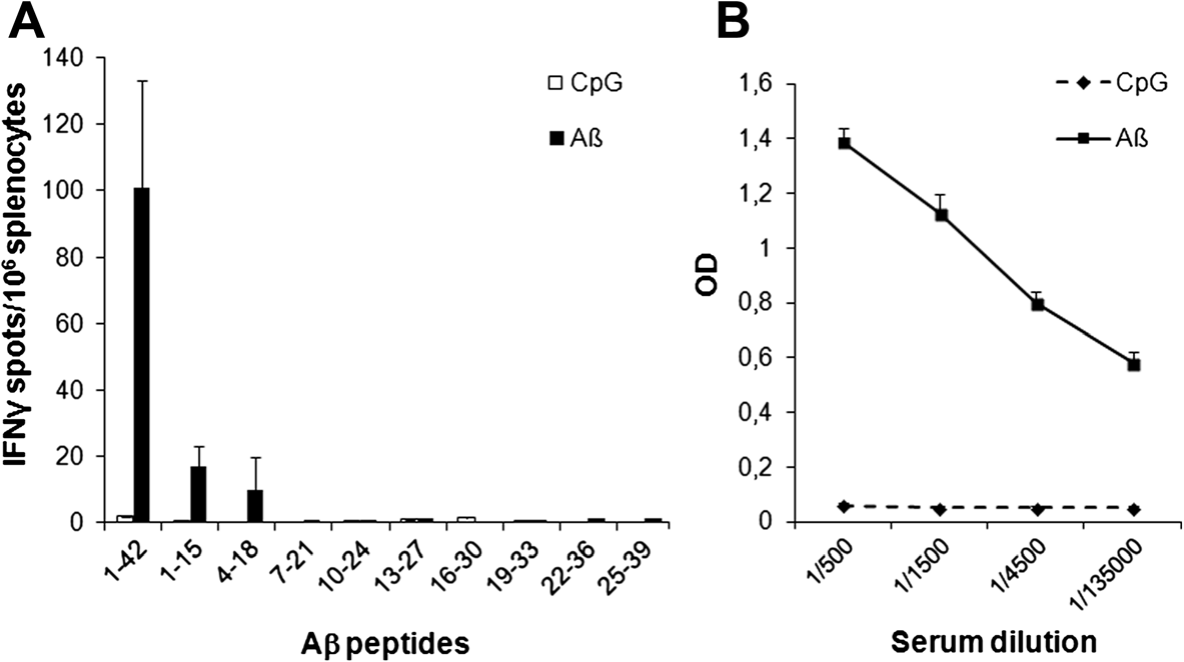
Aβ-specific CD4^+^ T cells and antibodies but not CD8^+^ T cells are triggered in HLA-A2.1/HLA-DR1 mice upon Aβ vaccination. **(a)** Frequency of IFN-γ secreting splenocytes in Aβ-immunized HLA-A2.1/HLA-DR1 mice, as assessed by ELISPOT. Splenocytes (10^6^/wells) from mice immunized with either PBS/CpG/IFA or Aβ1-42 in CpG/IFA were stimulated in triplicate for 18 h at 37 °C with Aβ1-42 (40 μg/ml) or 15-mer overlapping peptides (10 μg/ml) covering the full length of Aβ1-42. Results are presented as numbers of peptide-specific IFNγ-secreting cells per 10^6^ splenocytes, calculated after subtracting the mean number of spots obtained in the absence of peptide. **(b)** Serum levels of Aβ-specific antibodies were measured by ELISA using Aβ-coated plates. Data are expressed as relative OD. Mean ± SD (two to four mice/group). Results are representative of two independent experiments

Since T cell reactivity is strongly modulated by MHC genotype, we first examined whether Aβ1-42 comprises epitopes able to bind human MHC-I molecules. In silico analysis of binding affinities for a large number of MHC class I alleles (29 HLA-A or -B) of Aβ1-42-derived candidate nonamer peptides was carried out using BioInformatics and Molecular Analysis Section (BIMAS) predictive algorithm. Whereas a large proportion of alleles did not bind any Aβ-derived nonamer, about 30 % of tested alleles bound potential Aβ-derived epitopes with an affinity ranging from high to moderate (Table 1). Among these, HLA-A2.01 was found to potentially bind Aβ16-24 (BIMAS score: 453) and Aβ33-41 (BIMAS score: 15). These data indicate that Aβ can potentially promote the development of CD8^+^ T cell responses in selected MHC haplotypes. To assess this hypothesis, HLA-A2.1/HLA-DR1/H-2^b−/−^ double Tg mice on the C57BL/6 background (19–20) were immunized with Aβ/CpG/IFA. Analysis of splenocytes 14 days later showed that only CD4^+^ T cells that secrete IFNγ responded to Aβ1-42 immunization and recognized an immunodominant epitope located between amino acids 1–18 (Fig. 1a). Of note, Aβ28-42 15-mer candidate peptide could not be productively synthetized, likely due to its very high hydrophobicity and poor solubility. Although we cannot rule out that such peptide may include a potential MHC-II-restricted epitope, its biochemical features make unlikely the efficient MHC binding and presentation of Aβ28-42. Moreover, Aβ-specific antibodies were detected by ELISA in the serum of immunized mice (Fig. 1b). However, this immunization procedure did not trigger any CD8^+^ T cells able to recognize dendritic cells loaded with either Aβ or overlapping nonamer peptides covering the entire Aβ sequence (data not shown).

**Table 1:** Epitope prediction by BIMAS algorithm: estimated binding affinities of Aβ-derived nonamers to various HLA molecules

HLA molecule	Start AA position	Nonamer peptide	BIMAS score^a^
A1	20	VFFAEDVGS	18
A02.01	16	KLVFFAEDV	*453*
	33	GLMVGGVVI	15
A02.05	31	IIGLMVGGV	6
A24	9	GYEVHHQKL	*396*
A3	16	KLVFFAEDV	2
A11.01	-	*-*	-
A31.01	-	**-**	-
A33.02	7	DSGYEVHHQ	1.5
A68.01	24	VGSNKGAII	24
B14	4	FRHDSGYEV	4
B27.02	4	FRHDSGYEV	20
	2	AEFRHDSGY	15
B27.05	4	FRHDSGYEV	*600*
	2	AEFRHDSGY	*75*
	15	QKLVFFAED	27
	8	SGYEVHHQK	25
B28.01	-	**-**	-
B35.01	26	SNKGAIIGL	3
B37.01	-	**-**	-
B38.01	12	VHHQKLVFF	30
B39.01	-	**-**	-
B39.02	27	NKGAIIGLM	12
B40	2	AEFRHDSGY	*32*
	10	YEVHHQKLV	16
B44.03	2	AEFRHDSGY	*240*
B51.01	24	VGSNKGAII	*104*
	32	IGLMVGGVV	*57*
	28	KGAIIGLMV	24
B51.02	33	GLMVGGVVI	*132*
	24	VGSNKGAII	*88*
	28	KGAIIGLMV	28
B51.03	32	IGLMVGGVV	*52*
	24	VGSNKGAII	*48*
	28	KGAIIGLMV	*40*
B52.01	32	IGLMVGGVV	*300*
	24	VGSNKGAII	*75*
	28	KGAIIGLMV	*36*
B62	32	IGLMVGGVV	5
B7	26	SNKGAIIGL	4
B8	26	SNKGAIIGL	4

^a^Estimated half time of dissociation of corresponding MHC/peptide complexes, using BIMAS algorithm. Values above significant affinity threshold are depicted in italic

**Fig. 2:**
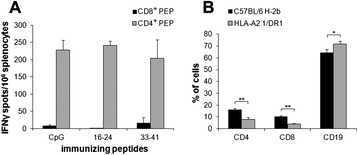
Immune responses of HLA-A2.1/HLA-DR1 mice after immunization with Aβ-derived CD8^+^ candidate epitopes. **(a)** Frequency of IFNγ-secreting splenocytes in peptide-immunized mice, as assessed by ELISPOT. Spleen cells (10^6^/wells) from mice immunized with either Aβ16-24 or Aβ33-41 in CpG/Padre/IFA or with PBS/CpG/Padre/IFA were stimulated in triplicate for 18 h at 37 °C with the immunizing peptide or Padre CD4^+^ helper peptide (10 μg/ml). Results are presented as numbers of peptide-specific IFN-γ-secreting cells per 10^6^ splenocytes, calculated after subtracting the mean number of spots obtained in the absence of peptide. **(b)** Phenotypic analysis of C57BL/6 wt and HLA-A2.1/HLA-DR1 mice. Percentage of splenocytes positive for CD4, CD8, and CD19 markers as measured by FACS*.* Mean ± SD (two to four mice/group). Results are representative of two independent experiments. Mann–Whitney U test, *p ≤ 0.05, **p ≤ 0.01

The two HLA-A2.01-restricted Aβ-derived epitopes, Aβ16-24 and Aβ33-41, were then mixed with a 14-mer human CD4^+^ helper epitope (Padre) in CpG/IFA and used for sc immunization in HLA-A2.1/HLA-DR1 mice. Padre (pan-DR epitope) was used in all experiments using HLA-DR transgenic mice, based on its very high MHC-II binding and helper activity [27]. The number of IFNγ-secreting splenocytes was high in response to the Aβ-unrelated CD4^+^ helper epitope but weak or null in response to the corresponding Aβ-derived nonamer CD8^+^ peptides (Fig. 2a). Phenotypic analysis revealed that the percentage of CD4^+^ and CD8^+^ T cells was twice lower in the spleen of untreated HLA-A.2.1/HLA-DR1 mice (7.8 and 4.9 %, respectively) than in regular C57BL/6 mice (16.4 and 10.4 %, respectively; *p* = 0.0003) (Fig. 2b). Such altered basal numbers of CD8^+^ T cells may contribute to the weak functional CD8^+^ T cell responses to Aβ vaccination in this mouse model. Altogether, these data suggest that Aβ-specific CD8^+^ T cell responses cannot be efficiently triggered in humanized HLA-A2.1/HLA-DR1/H-2^b−/−^ mice.

### Aβ-specific CD8^+^ T cells can be triggered in C57BL/6 mice by anchor-modified peptides

**Fig. 3:**
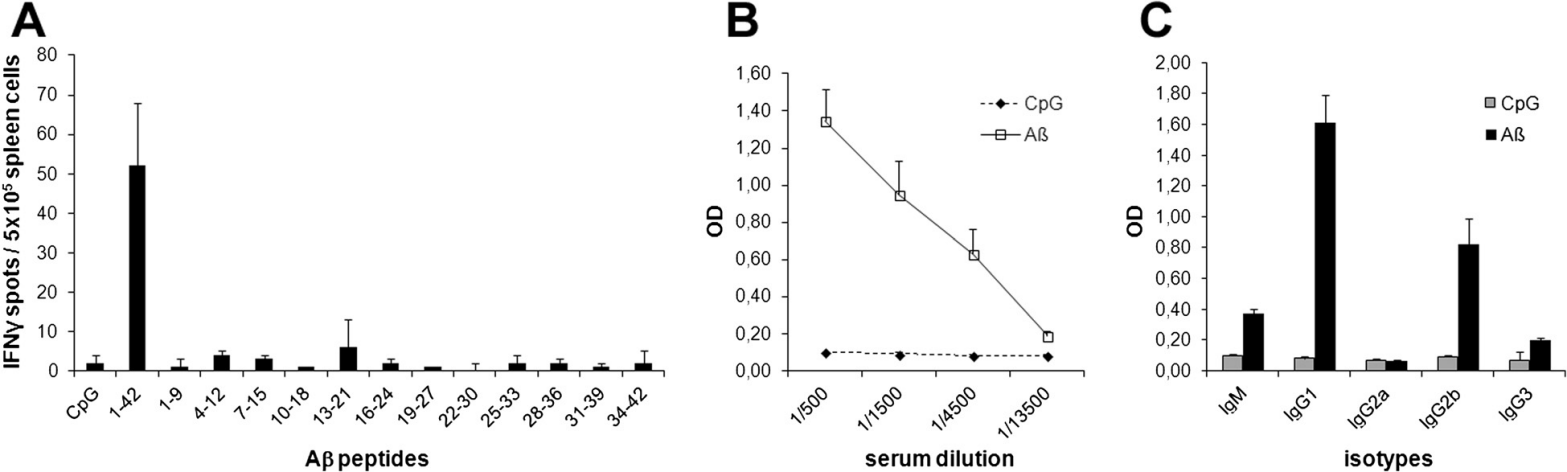
Analysis of Aβ-specific immune responses in regular C57BL/6 mice upon vaccination with Aβ1-42. **(a)** Frequency of Aβ-specific IFNγ-producing splenocytes in immunized mice, as assessed by ELISPOT. Spleen cells (10^6^/wells) from mice immunized with either PBS/CpG/IFA or Aβ1-42 in CpG/IFA were restimulated in triplicate for 18 h with Aβ1-42 (40 μg/ml) or a panel of overlapping nonamer peptides (10 μg/ml) covering the full length of Aβ1-42. Results are presented as numbers of peptide-specific IFNγ-secreting cells per 10^6^ splenocytes, calculated after subtracting the mean number of spots obtained in the absence of peptide. **(b, c)** Aβ-specific antibody responses in Aβ1-42-immunized mice. Levels of anti-Aβ antibodies in the sera were determined by ELISA using Aβ-coated plates. Data are expressed as relative OD. Mean ± SD (two to four mice/group). Results are representative of two independent experiments

In order to appropriately address the impact of Aβ-specific CD8^+^ T cell responses in vivo, we aimed at identifying Aβ-derived epitopes able to trigger specific CD8^+^ T cells in regular C57BL/6 mice (H-2^b^). Mice were immunized with Aβ/CpG/IFA, and splenocytes were analyzed 14 days later for the presence of Aβ-specific T cells. Although splenocytes secreted IFNγ in response to full-length Aβ1-42, none of the 12 overlapping Aβ-derived nonamer peptides reactivated effector cells (Fig. 3a). Antibodies specific for Aβ1-42 were detected in the serum of immunized mice (Fig. 3b) and were predominantly of IgG1 and IgG2b isotypes, suggesting the development of a Th2 type immune response (Fig. 3c). Of note, attempts to generate Aβ-specific CD8^+^ T cell responses using APP-encoding DNA also failed (data not shown). These results suggest that vaccination with full-length Aβ can efficiently elicit CD4^+^ but not CD8^+^ T cell responses in the H-2^b^ mouse haplotype, suggesting the poor immunogenicity of endogenously processed Aβ-derived nonamer peptides in this MHC context.

BIMAS predictive algorithm was used to identify Aβ-derived nonamer peptides bearing MHC-I-binding motifs in H-2^b^ haplotype. Two peptides were found to bind H-2-D^b^ molecules with moderate (Aβ23-31, score 45) and low (Aβ33-41, score 4) theoretical affinities (Table 2), and none were found to bind H-2-K^b^ molecules.

**Table 2:** Sequence and binding affinity of Aβ-derived H-2-D^b^-restricted candidate epitopes

Peptides	Amino acid sequences									BIMAS score^a^
Aβ23-31	D	V	G	S	N	K	G	A	I	45
23-31NP	*A*	*S*	*N*	S	*N*	K	G	A	*M*	286
Aβ33-41	G	L	M	V	G	G	V	V	I	4
33-41NP	*A*	*S*	*N*	V	*N*	G	V	V	*M*	343
NP366	*A*	*S*	*N*	E	*N*	M	D	A	*M*	286

Amino acid sequence of nonamer peptides derived from Aβ using the BIMAS prediction algorithm. Aβ peptides were modified (NP peptides) by replacing amino acids at the H-2-D^b^ anchor sites (depicted in italic) by amino acids from a high-binding affinity peptide (influenza virus nucleoprotein NP366)

^a^Estimated half time of dissociation of H-2-D^b^/peptide complexes, using BIMAS algorithm

**Fig. 4:**
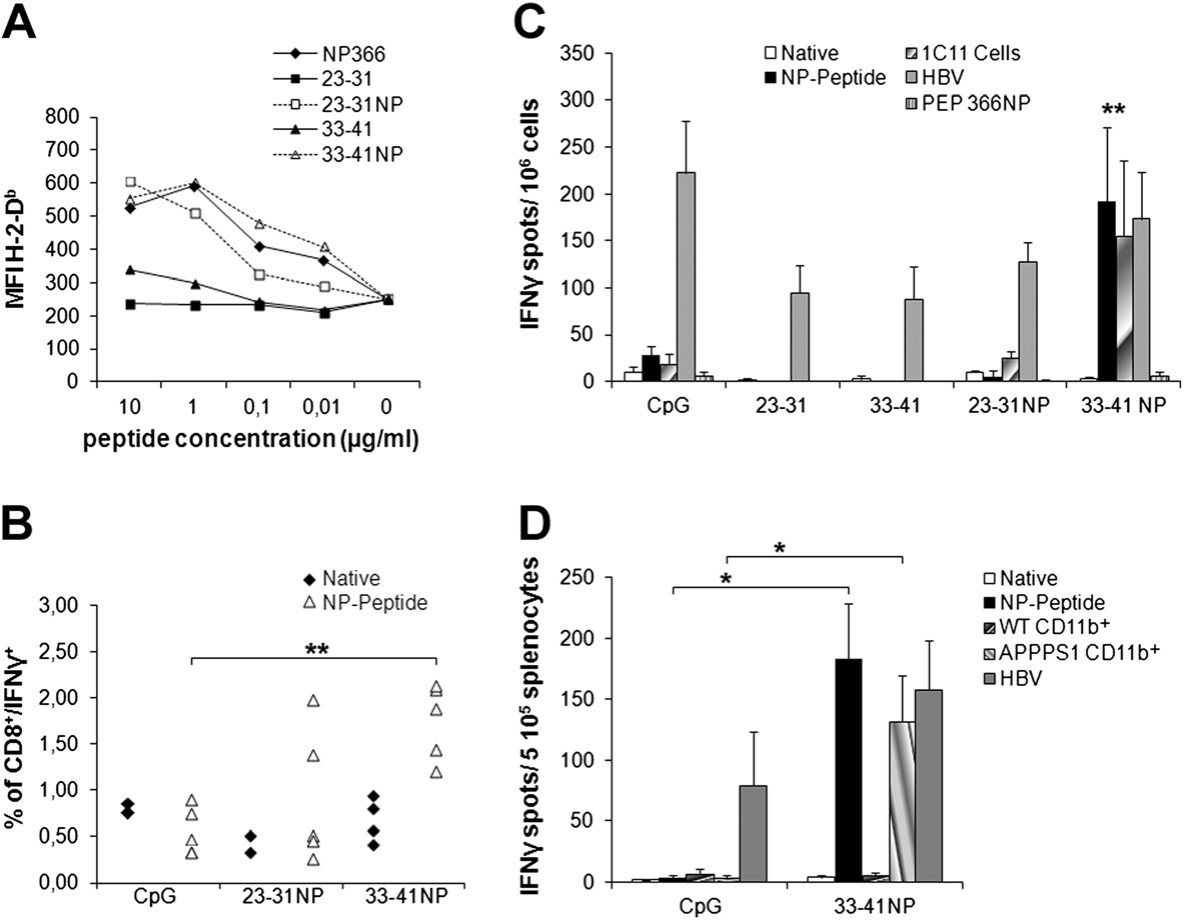
Binding affinities and immunogenicity of Aβ-derived CD8^+^ candidate epitopes. **(a)** RMAS cells were incubated with indicated concentrations of native or NP-modified peptides, and cell surface stabilization of H-2-D^b^ was evaluated by FACS. Data are presented as mean fluorescence intensity (MFI) of H-2-D^b^ staining. NP366 served as a high-affinity reference peptide. **(b)** Frequency of IFNγ-producing specific CD8^+^ T cells in the blood of immunized mice. C57BL/6 mice were vaccinated with NP-modified peptides in CpG/HBV/IFA or with PBS/CpG/HBV/IFA, and animals were bled 14 days later. Percentage of peptide-specific T cells among CD8^+^ cells in the blood was determined by intracellular IFNγ staining after in vitro restimulation. **(c, d)** Frequency of Aβ-specific IFNγ-producing splenocytes in immunized mice, as assessed by ELISPOT. (C) C57BL/6 mice were immunized with PBS/CpG/HBV/IFA or the indicated peptides in CpG/HBV/IFA, and 14 days later, spleen cells (10^6^/wells) were restimulated for 18 h with either the matching native or NP peptide, the HBV-derived helper peptide, or NP366 (10 μg/ml). For immunization with PBS/CpG/HBV/IFA, splenocytes were restimulated with each of the native or NP peptides and data were expressed as mean of the two values. Splenocytes were also stimulated with mitomycin-treated 1C11 cells (2 × 10^4^/wells), a neuronal cell line expressing both APP and H-2^b^ MHC-I. Results are presented as numbers of IFN-γ-secreting cells per 10^6^ splenocytes, after subtracting the mean number of spots obtained in the absence of peptide or cells. (D) Splenocytes (2 × 10^5^/wells) of C57BL/6 mice previously immunized with either PBS/CpG/HBV/IFA or 33-41NP mixed in CpG/HBV/IFA were restimulated on day 14 with 33-41NP (10 μg/ml) or CD11b^+^ microglia (3 × 10^4^/wells) isolated from the brain of WT or APPPS1 mice. Mean ± SEM (three to four mice/group). Results are representative of two independent experiments. Mann–Whitney U test, *p ≤ 0.05, **p ≤ 0.01

The candidate peptides were synthesized, and their actual affinity was determined by measuring their capacity to bind and stabilize H-2-D^b^ molecules on the cell surface of RMAS cells. Incubation with various peptide concentrations weakly stabilized MHC class I expression as compared to incubation with a reference peptide of high affinity (Influenza virus nucleoprotein, NP366, score 286) (Fig. 4a). To increase binding affinities of Aβ23-31 and Aβ33-41, amino acids at H-2-D^b^ anchor positions (P1, P2, P3, P5, and P9) were substituted by those of the high-affinity peptide NP366 (Table 2; NP peptides). These modifications highly increased the theoretical affinities of the two peptides (Aβ23-31NP, score 286; Aβ33-41NP, score 343) for H-2-D^b^ molecules and lead to MHC class I stabilization on RMAS cells to a similar level than with NP366 reference peptide (Fig. 4a).

C57BL/6 mice were immunized with native or NP-modified epitopes mixed with a 14-mer CD4^+^ helper peptide (I-A^b^-restricted murine epitope, HBV core) in CpG/IFA. The frequency of CD8^+^ T cells specific for the native or NP peptides was first evaluated in the blood by intracellular IFNγ staining assay. In vitro restimulation for 4 h with native peptides did not result in enhanced percentages of peripheral CD8^+^IFNγ^+^ cells in any groups of treated mice (Fig. 4b). In contrast, upon in vitro restimulation with the immunizing NP peptide, mice immunized with 33-41NP displayed significantly higher percentages of CD8^+^IFNγ^+^ cells than mice receiving CpG alone (1.78±0.19 versus 0.57±0.11, respectively, *p* = 0.01). Of note, no IFNγ^+^ cells were observed in CD8^−^ lymphocytes, confirming that 33-41NP selectively triggers CD8^+^ and not CD4^+^ T cell responses (data not shown). Frequency of CD8^+^IFNγ^+^ cells was not consistently enhanced in the blood of 23-31NP-immunized mice, although two mice out of five had a detectable IFNγ response (Fig. 4b). The magnitude and specificity of vaccine-induced immune responses was also measured by ELISPOT assay 14 days post immunization. Results indicated that splenocytes from 33-41NP-immunized mice but not splenocytes from mice immunized with the 33–41 native peptide significantly (*p* < 0.01) secreted IFNγ in response to the NP-modified but not to the native peptide. Notably, 33-41NP-specific splenocytes also did not respond to the NP366 peptide, confirming that they were specific for TCR-exposed residues from Aβ33-41 (Fig. 4c). In contrast, the number of IFNγ-secreting splenocytes was not significantly enhanced in mice immunized with either Aβ23-31 or Aβ23-31NP (Fig. 4c). IFNγ-producing cells were also detected in splenocytes from 33-41NP-immunized mice upon restimulation with 1C11 neuronal cells, which express both APP and H-2^b^ MHC-I but not MHC-II molecules (Fig. 4c). Similar results were obtained with 1C11 cells when using CD8^+^ T cells purified from 33-41NP-immunized splenocytes (data not shown). Importantly, splenocytes from 33-41NP-immunized mice significantly (*p* < 0.01) secreted IFNγ in response to brain-derived CD11b^+^ microglial cells isolated from APPPS1 mice but not from WT animals (Fig. 4d). Altogether, these results indicate that Aβ33-41 epitope can be naturally processed and presented in both microglia and neuronal cells, and that 33-41NP peptide efficiently stimulates an Aβ-specific CD8^+^ T cell repertoire in C57BL/6 mice.

**Fig. 5:**
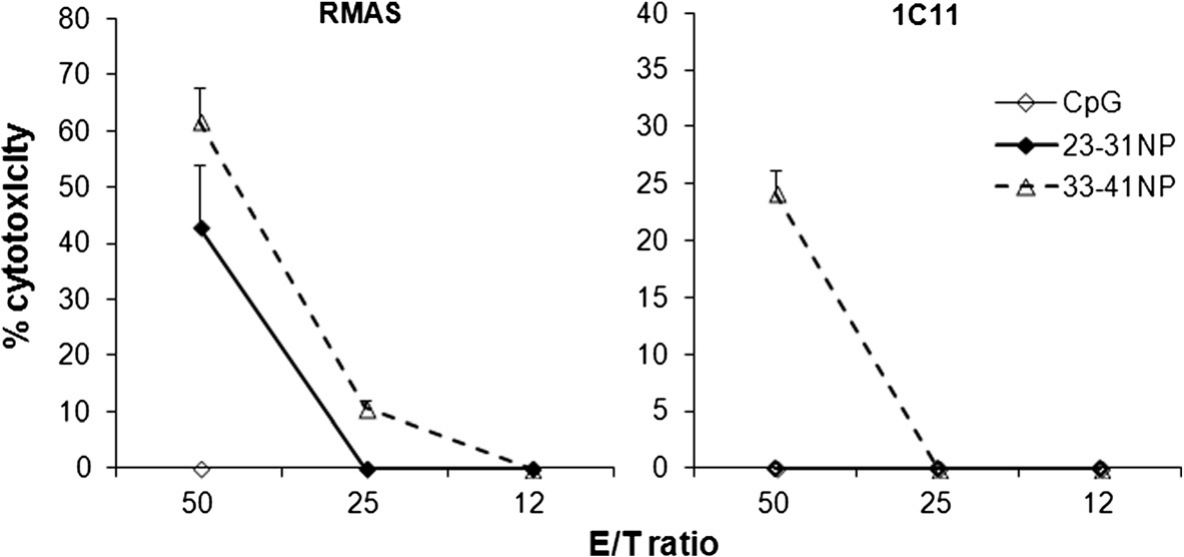
Immunization of C57BL/6 mice with NP peptides triggers specific and functional cytotoxic cells. Spleen cells from mice immunized with PBS/CpG/HBV/IFA or with the indicated NP peptides mixed in CpG/HBV/IFA were restimulated for 5 days with matching NP peptides (10 μg/ml). In the case of immunization with PBS/CpG/HBV/IFA, splenocytes were restimulated with each of the NP peptides. Various concentrations of immune effectors specific for 23-31NP or 33-41NP were incubated for 4 h in the presence of ^51^Cr-labeled peptide-loaded RMAS cells (10^5^ cells/wells) or of ^51^Cr-labeled 1C11 cells (2 × 10^4^/wells). In the case of immunization with PBS/CpG/HBV/IFA, splenocytes were incubated with one or the other peptide-loaded RMAS cells or medium alone. Results are reported as the percentage of cytotoxicity calculated as described in Methods section. Results are representative of two independent experiments

We next analyzed the cytolytic activity of splenocytes from NP-peptide-immunized mice. After a 5-day in vitro restimulation in the presence of 23-31NP or 33-41NP, cytotoxic activity towards ^51^Cr-labeled peptide-loaded RMAS cells or 1C11 neuronal cells was evaluated. Immune effectors specific for 23-31NP or 33-41NP were able to lyse RMAS targets loaded with the matching NP peptides (50.7 and 65.9 % lysis, respectively; E:T ratio 1:50) (Fig. 5a). However, only splenocytes from 33-41NP-immunized mice moderately lysed (23.3 %) 1C11 cells (Fig. 5b). Thus, we identified one epitope, Aβ33-41, which is restricted to H-2-D^b^ MHC class I molecule and is naturally processed in both microglia and neuronal cells. When anchor optimized, modified Aβ33-41 peptide can elicit anti-Aβ CD8^+^ T cell responses that display cytotoxic activity towards Aβ-expressing neuronal target cells.

### Aβ33-41NP activates specific CD8^+^ T cells in APPPS1 mouse model of AD

**Fig. 6:**
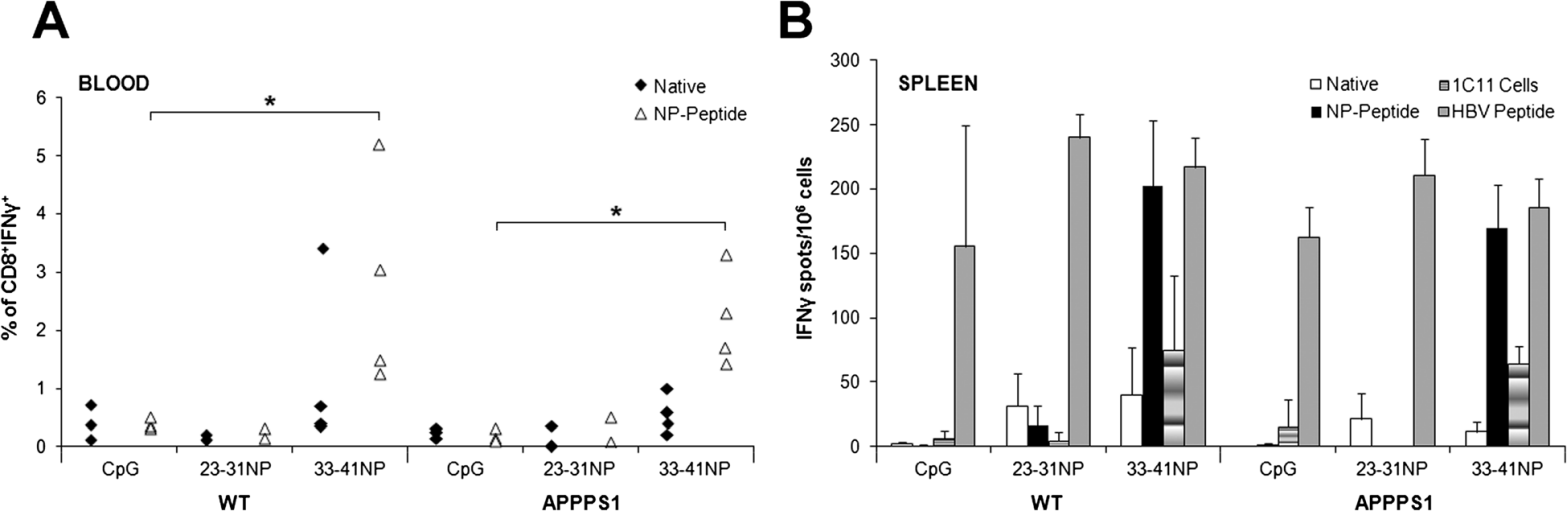
Analysis of Aβ-specific immune responses in APPPS1 mice upon vaccination with Aβ-derived nonamer epitopes. APPPS1 mice or WT littermates were immunized with indicated native or NP-modified peptides mixed in CpG/HBV/IFA or with PBS/CpG/HBV/IFA alone. **(a)** Frequency of peptide-specific IFNγ-producing cells among CD8^+^ lymphocytes in the blood was determined 14 days after vaccination, by intracellular IFNγ staining after restimulation with the native or NP peptides. **(b)** Frequency of Aβ-specific IFNγ-secreting splenocytes in immunized mice, as assessed by ELISPOT. Fourteen days after vaccination, spleen cells (10^6^/wells) were restimulated in triplicate for 18 h with either the matching native or NP peptide or with HBV-derived helper peptide (10 μg/ml). In the case of immunization with PBS/CpG/HBV/IFA, splenocytes were restimulated with each of the native or NP peptides and data are expressed as the mean of the two values obtained for each category of peptide (i.e., native or NP). Splenocytes from immunized mice were also stimulated in vitro with mitomycin-treated 1C11 cells (2 × 10^4^/wells). Results are presented as numbers of IFN-γ-secreting cells per 10^6^ splenocytes, calculated after subtracting the mean number of spots obtained in the absence of peptide or cells. Mean ± SD (three to four mice/group). Results are representative of two independent experiments. Mann–Whitney U test, *p ≤ 0.05

The H-2-D^b^-restricted Aβ-derived epitopes we identified were used to elicit Aβ-specific cytotoxic CD8^+^ T cell responses in the C57BL/6-based APPPS1 mouse model of AD [22]. APPPS1 mice and WT littermates were immunized between the age of 4 to 7 months with 23-31NP or 33-41NP mixed with CD4^+^ helper peptide (HBV core) in CpG/IFA. The frequency of peptide-specific CD8^+^ T cells was measured in the blood 12–14 days post immunization. Results depicted in Fig. 6a show that a significant percentage of CD8^+^IFNγ^+^ cells was observed in the blood of both APPPS1 and WT mice immunized with 33-41NP (*p* < 0.01) but not in 23-31NP-immunized mice, compared to mice receiving only CpG. Whereas such responses were observed upon in vitro restimulation with NP peptides, only low and non-significant responses were detected after restimulation with the native peptides. ELISPOT assay further showed that immunization with 33-41NP but not with 23-31NP was able to elicit in vivo Aβ-specific IFNγ-secreting splenocytes in both APPPS1 mice and WT littermates. These cells recognized in vitro the matching NP peptide or the endogenously processed epitope in 1C11 neuronal cells but did not recognize the native peptide when loaded exogenously (Fig. 6b). Of note, immunized APPPS1 and WT mice similarly responded to the Aβ*-*unrelated CD4^+^ helper epitope. These results confirm that Aβ33-41NP was able to activate peripheral Aβ-specific CD8^+^ T cells in APPPS1 mice.

### Aβ33-41NP specifically promotes CD8^+^ T cell infiltration into the brain of APPPS1 mice without triggering encephalitis

**Fig. 7:**
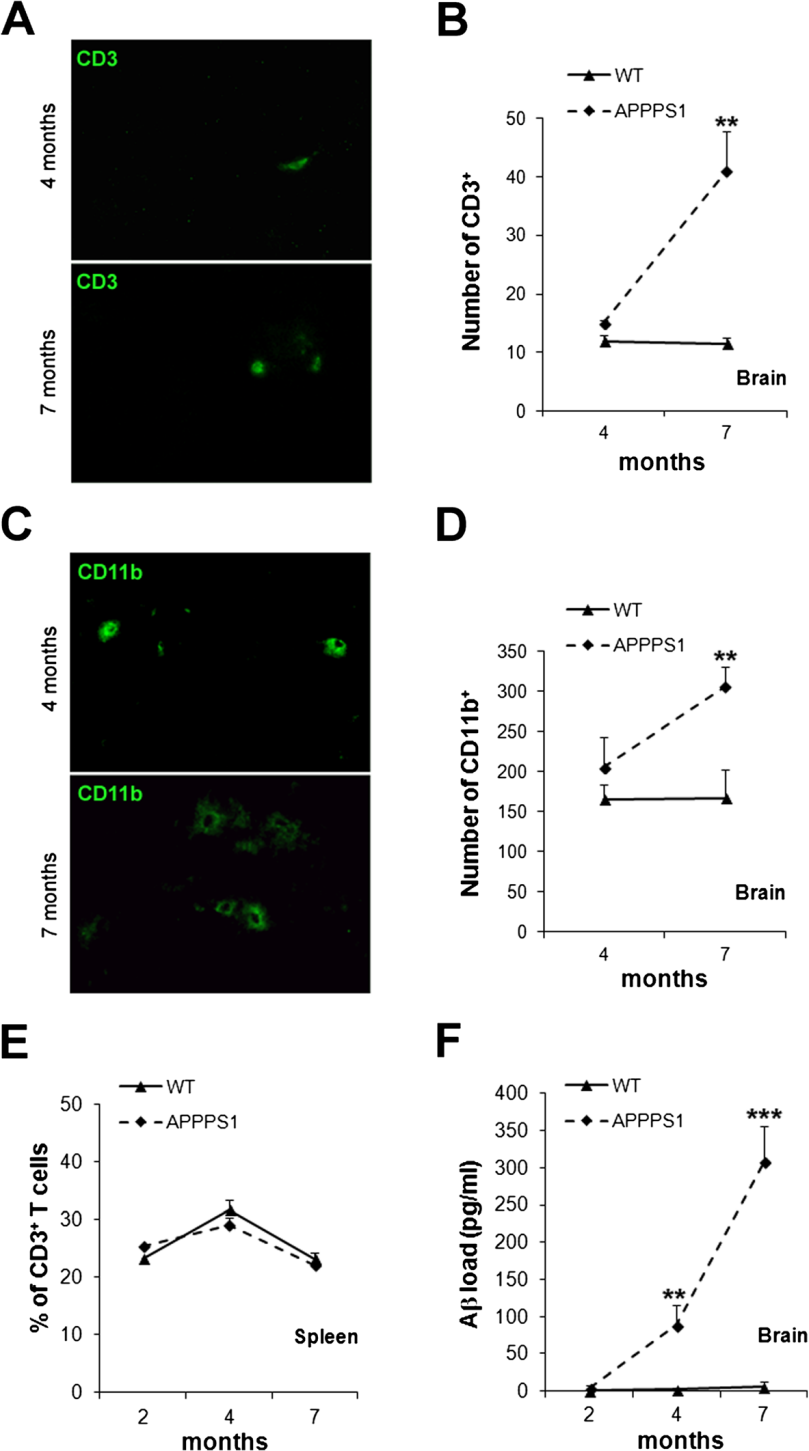
Comparative analysis of immune populations in APPPS1 and WT mice between 4 and 7 months of age. Representative images from brain cortex of APPPS1 mice **(a, c)** and numbers of CD3^+^ T cells **(b)** or CD11b^+^ cells **(d)** in the brain parenchyma of WT and APPPS1 mice. **(e)** Percentage of CD3^+^ T cells in the spleen analyzed by flow cytometry. **(f)** Quantification by ELISA of Aβ load in the brain. Mean ± SD (three to five mice/group). Results are representative of two independent experiments. Mann–Whitney U test, **p ≤ 0.01, ***p ≤ 0.001

The magnitude of T cell infiltration and CD11b immunoreactivity in the brain was evaluated as a function of age in APPPS1 as compared to WT mice. Immunohistochemistry analyses showed that the number of CD3^+^ T cells significantly increased (*p* = 0.01) in the brain of APPPS1 mice between 4 and 7 months of age. In contrast, only moderate T cell infiltration was observed with aging in the brain of WT littermates (Fig. 7a, b). The number of CD11b^+^ cells remains constant in the brain of WT mice, whereas it significantly increases (*p* = 0.02) between 4 and 7 months of age in APPPS1 mice (Fig. 7c, d), in line with the described microgliosis associated with Aβ deposition in this model [22]. The progressive accumulation of CD3^+^ and CD11b^+^ cells in the brain of APPPS1 mice parallels the significant (*p* = 0.001) Aβ deposition measured by ELISA (Fig. 7f). Of note, no differences in the percentages of CD3^+^ cells were detected in the spleen of APPPS1 and WT mice whatever the age (Fig. 7e). These observations suggested that recruitment and/or local accumulation of CD3^+^ T cells and CD11b^+^ mononuclear phagocytes in the brain of APPPS1 mice is related to disease development.

**Fig. 8:**
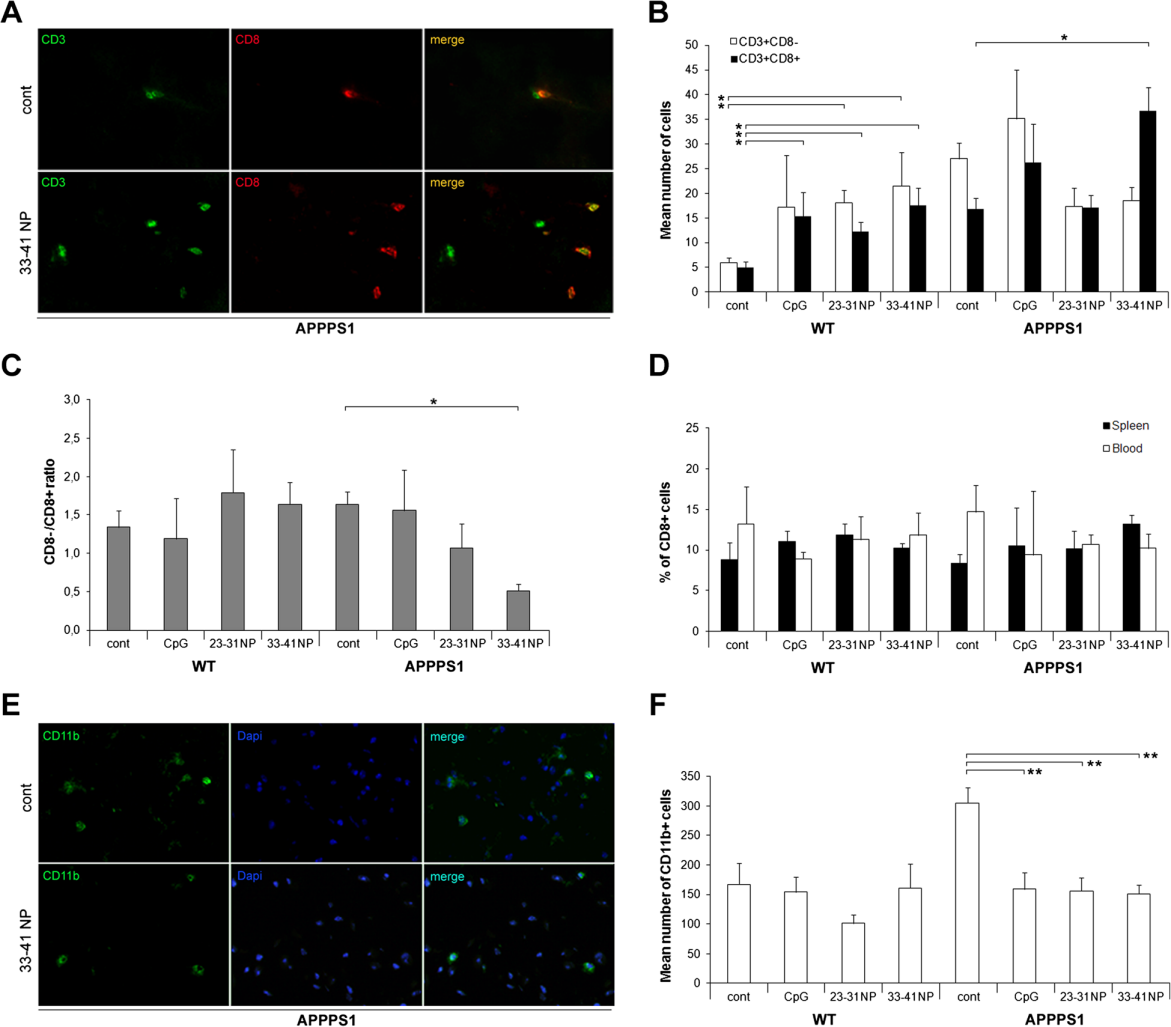
Aβ33-41NP specifically promotes CD8^+^ T cell infiltration into the brain of APPPS1 mice. Four-month-old APPPS1 mice or WT littermates were immunized twice with 23-31NP or 33-41NP mixed in CpG/HBV/IFA, or treated with PBS/CpG/HBV/IFA alone. Brains were harvested between 6 and 7 month of age, and immune cell infiltration was analyzed by immunohistochemistry. Brain sections were stained with anti-CD3 + anti-CD8 mAbs or anti-CD11b mAb. **(a)** Representative images from brain cortex of control or 33-41NP-immunized APPPS1 mice. **(b)** Numbers of CD3^+^CD8^−^ or CD3^+^CD8^+^ cells per brain section. **(c)** CD8^−^/CD8^+^ ratio. **(d)** Percentage of CD8^+^ cells in the blood and spleen analyzed by flow cytometry. **(e)** Representative images of CD11b labeling in the cortex of control or 33-41NP-immunized APPPS1 mice. **(f)** Numbers of CD11b^+^ cells per brain section. Mean ± SEM (three to five mice/group). Results are representative of two independent experiments. Mann–Whitney U test, *p ≤ 0.05, **p ≤ 0.01

To evaluate if peripherally activated Aβ-specific CD8^+^ T cells specifically infiltrate the brain upon vaccination, APPPS1 and WT mice were immunized twice at 1-month interval with either 33-41NP or 23-31NP as a control peptide. Cells positive for CD3 and/or CD8 markers were then quantified in the brain 2–3 weeks after the last immunization. Higher numbers of CD3^+^ T cells previously observed in the brain of untreated APPPS1 mice as compared to age-matched WT littermates were related to an increase in both CD8^−^ (*p* = 0.018) and CD8^+^ (*p* = 0.04) T cells. Importantly, CD8^−^ cells always predominate over CD8^+^ cells in both APPPS1 and WT mice (Fig. 8a, b). A significantly enhanced infiltration (*p* < 0.01) of both CD3^+^CD8^−^ and CD3^+^CD8^+^ cells was detected into the brain of WT mice following immunization with CpG + CD4^+^ helper epitope, whether or not it was mixed with the CD8^+^ peptides (Fig. 8b). In contrast, the number of CD3^+^CD8^+^ cells selectively increased in the brain parenchyma of APPPS1 mice after vaccination with 33-41NP (36.7+/−4.8; *p* = 0.057) but not with 23-31NP (17.1+/−2.5) as compared to untreated animals (16.8+/−2.2) (Fig. 8a, b). The number of CD3^+^CD8^−^ cells decreased in the brain of APPPS1 mice immunized with 33-41NP (18.6+/−2.6) and 23-31NP (17.3+/−3.7), although not significantly as compared to untreated mice (27.0+/−3.2). Of note, numbers of both CD3^+^CD8^−^ and CD3^+^CD8^+^ cells are slightly increased after CpG treatment alone. Analysis of CD8^−^/CD8^+^ ratios confirmed that CD3^+^CD8^−^ cells predominate over CD3^+^CD8^+^ cells into the brain in all immunizing conditions including CpG alone, except for APPPS1 mice immunized with 33-41NP (CD8^−^/CD8^+^ = 0.57) (Fig. 8c). No significant differences in the overall percentage of CD8^+^ cells were observed in either the spleen or blood of WT and APPPS1 mice after any type of immunizations, suggesting a brain-restricted enhancement of CD8^+^ T cell recruitment in 33-41NP-immunized APPPS1 mice (Fig. 8d).

Variations in the number of CD11b^+^ cells upon immunization were also evaluated in the brain of WT and APPPS1 mice (Fig. 8e, f). In accordance with Aβ-related microgliosis, untreated APPPS1 mice display significantly higher numbers of CD11b^+^ cells than WT mice. Of note, whereas immunization with CpG, 23-31NP, or 33-41NP induced no modification in WT mice, it significantly reduces (*p* < 0.01) the number of CD11b^+^ mononuclear phagocytes in the brain parenchyma of APPPS1, leading to similar numbers as in age-matched WT mice (Fig. 8e, f).

Importantly, despite peripheral activation of Aβ-specific CD8^+^ cytotoxic effectors and enhanced infiltration of CD8^+^ T cells into the brain of Aβ33-41NP-immunized APPPS1 mice, no clinical signs (e.g., kyphosis, paraplegia) of severe autoimmune neuroinflammation were observed. Altogether, these results suggest that vaccine-induced Aβ33-41NP-specific CD8^+^ T cells preferentially and selectively accumulate into the brain parenchyma of APPPS1 mice at later disease stages associated with increased Aβ deposition, without triggering encephalitis nor severe brain damages.

## Discussion

The development of meningoencephalitis in some AD patients following Aβ vaccination (AN1792) was sought to involve pro-inflammatory CD4^+^ T cells [7, 8]. In this work, using mouse models, we address the possible implication of auto-aggressive Aβ-specific CD8^+^ T cells in the development of these severe side effects. In contrast to MHC-II-restricted epitopes, the length of peptides able to bind MHC-I molecules is precisely determined by biochemical constraints. Consequently, the array of potential MHC-I-restricted epitopes derived from a small Ag like Aβ1-42 is rather limited, which might explain why only 6 % of patients developed severe adverse effects following Aβ vaccination [6]. In silico analyses using BIMAS predictive algorithm suggested that Aβ might give rise to MHC-I-restricted epitopes in approximately 30 % of class I HLA alleles, including HLA-A2.1 (Table 1). We first sought to validate in HLA-Tg mice the capacity of such Aβ-derived epitopes to recruit CD8^+^ T cell responses. Yet, HLA-A2.1/HLA-DR1/H-2^b−/−^ double transgenic mice on C57BL/6 background failed to develop a functional response after immunization with Aβ16-24 candidate epitope, despite its theoretical high binding affinity for HLA-A2.1. Lack of CD8^+^ T cell response induction may result from altered overall frequency of CD4^+^ and CD8^+^ T cells in HLA-transgenic mice, lack of natural processing of this HLA-A2.1-restricted Aβ-derived epitope, or deletion of high-avidity T cells specific for dominant epitopes derived from a self-antigen.

Similarly, no Aβ-specific CD8^+^ T cell response was obtained in regular C57BL/6 mice upon vaccination with Aβ. In contrast to our prediction studies for class I HLA alleles, in silico predictive analyses revealed poor affinities of Aβ-derived H-2-D^b^-restricted epitopes. A specific approach aimed at optimizing the binding affinity and immunogenicity of these MHC-I-restricted candidate epitopes was thus required for accurately assessing the impact of Aβ-specific CD8^+^ T cells in response to vaccination. Optimized epitopes were engineered as previously described in various models [24, 28, 29]. Main MHC-I anchor residues of candidate epitopes were substituted by anchor residues of the NP366 reference peptide, which displays high affinity for H-2-D^b^, without altering TCR contact residues. Importantly, T-cell responses generated using such engineered epitopes retained their specificity for the matching native epitopes and did not recognize the reference peptide. Immunization with two such anchor-optimized epitopes, Aβ33-41NP and Aβ23-31NP, activated CD8^+^ T cells that secrete IFNγ and are cytotoxic towards peptide-loaded targets. However, only Aβ33-41 but not Aβ23-31 appears to be naturally processed and presented to immune effectors by a neuronal cell line expressing both APP and H-2^b^ MHC-I molecules [25, 26] as well as by brain-derived CD11b^+^ microglia isolated from APPPS1 mice. Of note, although neuronal cells, microglia, or other APCs such as dendritic cells (data not shown) efficiently present the endogenous peptide, Aβ33-41 native peptide could not restimulate Aβ33-41NP-specific CD8^+^ T cells when loaded exogenously. These data suggest that full endogenous machinery for antigen processing and presentation, including chaperone activities, is critically required for efficient presentation of potential low-affinity cryptic epitopes from pathogenic antigens. In contrast, when provided exogenously, such weak epitopes are likely very poorly efficient at replacing antigenic peptides from pre-formed MHC/peptide complexes at cell surface.

Immunization of APPPS1 mice with the anchor-optimized Aβ33-41NP peptide efficiently stimulated Aβ-specific CD8^+^ T cells in such C57BL/6-based mouse model of AD. Importantly, similar numbers of IFNγ-secreting CD8^+^ T cells were observed in the periphery of both APPPS1 and WT littermates following vaccination, ruling out an overall immunosuppressive environment in the context of AD. Our study does not confirm previous reports suggesting that APP overexpression in transgenic mice might be associated with impaired Aβ-specific adaptive immunity [30]. In contrast, our data are consistent with studies showing increased frequencies of Aβ-reactive T cells in the blood of both healthy elderly and patients with AD, as compared to younger healthy adults [12].

Several studies reported that the brain is continuously monitored by memory T cells, whereas only few naïve CD3^+^ T cells gain access to the brain in healthy individuals [31]. We found that numbers of CD3^+^ T cells significantly increase with age in the brain of APPPS1 mice. This augmentation reflected enhanced infiltration of both CD8^−^ and CD8^+^ populations, with a predominance of CD8^−^ over CD8^+^ cells. In the same line, increased numbers of T cells have been described in the brain parenchyma of AD patients, mostly in the hippocampus [32]. Importantly, inverted CD8^−^/CD8^+^ ratio with a predominance of CD8^+^ over CD8^−^ cells was selectively observed in the brain of APPPS1 but not WT mice after Aβ33-41NP immunization. These data suggest that such intracerebral accumulation of CD8^+^ T cells specifically occurs in the context of AD and depends on specific antigen expression, as supported by the capacity of brain-derived CD11b^+^ microglia from APPPS1 but not from WT mice to activate Aβ33-41NP-specific effectors. The preferential recruitment of Aβ-specific CD8^+^ T cells into the brain parenchyma of vaccinated APPPS1 mice is further supported by the fact that 1) induction of CD8^+^ T cell responses specific for Aβ23-31 peptide, which turned out not to be naturally processed, did not induce any change in the CD8^−^/CD8^+^ ratio into the brain of APPPS1 mice; 2) no modification in the overall percentages of CD4^+^ and CD8^+^ T cells was observed in the blood and spleen of APPPS1 mice after immunization. Altogether, these results suggest that Aβ-specific CD8^+^ T cells triggered by vaccination with anchor-modified epitope are specifically recruited into the brain parenchyma of APPPS1 mice. A mechanism of Ag-dependent recruitment of CD8^+^ T cells into the brain of EAE mice was previously described [33]. Our results are in line with these studies and suggest that Aβ-derived MHC-I-restricted epitopes and particularly Aβ33-41 may efficiently be processed and presented by cerebral vascular endothelial cells in the brain of AD mice.

Strikingly, despite specific infiltration of the brain by functional self-reactive CD8^+^ T cells and specific antigen processing and presentation by both neuronal cells and CD11b^+^ microglia, no clinical sign of autoimmune neuroinflammation or CNS damages was observed in Aβ33-41NP-immunized APPPS1 mice. In accordance with our data, increased numbers of CD3^+^ cells in the hippocampus of AD patients did not parallel activation of microglia nor disease stages. T cells were found negative for CD25 and the vast majority of them was CD45RO^+^ but CR3^−^, indicating that they were activated and potent cytokine producers but not fully differentiated, possibly due to insufficient stimuli or immunosuppressive environment in the brain [32]. Weak expression of MHC class I molecules on neurons [34] and/or their resistance to perforin-mediated lysis by CTLs [35] may partially account for such absence of detrimental effect of infiltrating CD8^+^ T cells. In addition, the CNS parenchyma was described to constitute a particular microenvironment with potent immunoregulatory properties, especially in the context of neuroinflammatory conditions such as AD. Astrocytes, activated microglia, and neurons can express several factors that negatively regulate T cell activation, proliferation, and cytokine production [36]. Moreover, it has been proposed that upon extravasation into the CNS, T cells primed in the periphery need to be restimulated by perivascular and/or meningeal APCs for being fully licensed before accessing the brain parenchyma [37]. The efficiency of these particular APCs at taking up Aβ and cross-presenting Aβ-derived epitopes to CD8^+^ T cells remains unknown and may also constitute a critical parameter restraining the functionality of Aβ-specific CD8^+^ T cells inside the brain parenchyma. Finally, we cannot rule out that altered behavior of infiltrating Aβ-specific CD8^+^ T cells in such mouse models may rely on other specific mechanisms related to migration and functional regulation of T cells within the AD brain parenchyma, which may significantly differ between mouse and human. In this line, a critical role of IFNγ in the migration and functional outcome of Aβ-specific CD4^+^ T cells in mouse models of AD has previously been discussed [38]. Deciphering the overall impact of such parameters on both CD4^+^ and CD8^+^ T cell responses in the context of the AD brain definitely requires further analyses. Such studies may hold the key for the development of refined mouse models of AD with optimized disease-related immunological relevance, which are of paramount importance for better understanding the pathophysiology of AD and for accurately evaluating the efficiency and safety of innovative immunotherapy approaches.

After both Aβ-specific or non-specific vaccination, the high number of CD11b^+^ cells observed in the brain of untreated APPPS1 mice was significantly reduced to similar levels than in WT littermates. In line with recent reports, these data suggest that modulation of peripheral immunity and inflammatory status may significantly impact the magnitude and/or shaping of parenchymal neuroinflammation, including modifications in microglia phenotype and activation profile [39, 40].

In conclusion, our results do not support a role for Aβ-specific CD8^+^ T cells in the development of meningoencephalitis after Aβ immunization. Whereas functional anti-Aβ CD8^+^ cytotoxic T cells can be peripherally activated and specifically recruited to the brain, immune ignorance or altered functionality of these effectors in the microenvironment of the AD brain parenchyma may be responsible for their lack of detrimental effect.

## Abbreviations

Aβ: amyloid-β peptide
AD: Alzheimer’s disease
APP: amyloid precursor protein
BIMAS: BioInformatics and Molecular Analysis Section
HBV: hepatitis B virus core peptide
mAb: monoclonal antibody
Tg: transgenic
WT: wild type
